# Neurodevelopmental domain characteristics and their association with core symptoms in preschoolers with autism spectrum disorder in China: a nationwide multicenter study

**DOI:** 10.1186/s12888-022-04028-5

**Published:** 2022-06-13

**Authors:** Qian Zhang, Qiu Li, Ting Yang, Li Chen, Ying Dai, Hua Wei, Ke Wang, Feiyong Jia, Lijie Wu, Yan Hao, Ling Li, Jie Zhang, Xiaoyan Ke, Mingji Yi, Qi Hong, Jinjin Chen, Shuanfeng Fang, Yichao Wang, Qi Wang, Chunhua Jin, Jie Chen, Tingyu Li

**Affiliations:** 1 Department of Child Health Care, Children’s Hospital of Chongqing Medical University, National Clinical Research Center for Child Health and Disorders, Ministry of Education Key Laboratory of Child Development and Disorders, Chongqing Key Laboratory of Child Health and Nutrition, Chongqing, 400014 China; 2 Children’s Medical Big Data Intelligent Application Chongqing University Engineering Research Center, Children’s Hospital of Chongqing Medical University, Chongqing, 400014 China; 3 Department of Developmental and Behavioral Pediatric, The First Hospital of Jilin University, Changchun, 130021 China; 4 Department of Children’s and Adolescent Health, Public Health College of Harbin Medical University, Harbin, 150081 China; 5 Department of Pediatrics, Tongji Hospital, Tongji Medical College, Huazhong University of Science and Technology, Wuhan, 430030 China; 6 Department of Children Rehabilitation, Hainan Women and Children’s Medical Center, Haikou, 570100 China; 7 Xi’an Children’s Hospital, Xi’an, 710003 China; 8 Child Mental Health Research Center of Nanjing Brain Hospital, Nanjing, 210013 China; 9 Department of Child Health Care, The Affiliated Hospital of Qingdao University, Qingdao, 266003 China; 10 Maternal and Child Health Hospital of Baoan, Shenzhen, 518133 China; 11 Department of Child Healthcare, Shanghai Children’s Hospital, Shanghai Jiao Tong University, Shanghai, 200040 China; 12 Children’s Hospital Affiliated to Zhengzhou University, Zhengzhou, 450053 China; 13 NHC Key Laboratory of Birth Defect for Research and Prevention, Hunan Provincial Maternal and Child Health Care Hospital, Changsha, 410008 China; 14 Deyang Maternity & Child Healthcare Hospital, Deyang, 618000 Sichuan China; 15 Department of Children Health Care, Capital Institute of Pediatrics, Beijing, 100020 China

**Keywords:** Autism, Preschool children, Functional development, Core symptoms, Multicenter

## Abstract

**Background:**

Autism spectrum disorder (ASD) is a group of clinically heterogenic neurodevelopmental disorders, with intellectual disability being one of its common comorbidities. No large-sample, multicenter study has focused on the neurodevelopmental aspect of preschoolers with ASD. This study investigated the neurodevelopmental characteristics of preschoolers with ASD in China and explored the association between them and the core symptoms.

**Methods:**

We enrolled 1019 ASD preschoolers aged 2–7 years old from 13 cities around China between May 2018 and December 2019, and used the revised Children Neuropsychological and Behavior Scale (CNBS-R2016) to assess their neurodevelopment. Their autistic core behaviors were evaluated based on their Social Responsiveness Scale (SRS), Autism Behavior Checklist (ABC), Child Autism Rating Scale (CARS), and communication warning behavior (CWB) scores in the CNBS-R2016.

**Results:**

Based on general developmental quotient (GQ) < 70, 68.4% of the preschoolers with ASD had a developmental delay (DD), rated mild in 32.7% of them. The highest DD rate (> 70%) was found in language and personal-social skills, followed by fine motor skills (68.9%). Gross motor skills had the lowest DD rate (34.0%). We found that fine motor, language, and personal-social developmental quotients (DQs) were significantly lower than gross motor skills in no DD (GQ > 70), mild DD (GQ 55–69), and moderate and below DD groups (GQ ≤ 54). Furthermore, the DQs for language and personal-social skills were significantly lower than for gross and fine motor skills in both DD groups. The ABC, SRS, CARS, and CWB scores in the no DD group were the lowest, moderate in the mild DD group, and highest in the moderate and below DD group. Besides, negative correlations were found between the DQs of the four domains and the ABC, SRS, CARS, and CWB scores, of which the language and personal-social skills DQs had the strongest correlations.

**Conclusions:**

Preschoolers with ASD had unbalanced neurodevelopment domain patterns and their neurodevelopmental levels were negatively correlated with the autism core symptoms. Hence, pediatricians should actively evaluate the neurodevelopment of children with ASD and conduct long-term follow-up during their early childhood to promote early diagnosis and develop personalized intervention plans.

**Trial registration:**

ChiCTR2000031194, registered on 03/23/2020.

## Background

Autism spectrum disorder (ASD) is a group of neurodevelopmental disorders characterized by social communication deficits, restricted interest in the surroundings, and stereotyped repetitive behaviors [1]. The reported ASD prevalence in the US was 2.3% in 8-year-old children in 2018 [2]. A National Surveys in China from 2013 found a prevalence of about 0.7% in children aged 6–12 years [3]. Such high prevalence rates attracted worldwide attention. Many individuals with ASD have accompanying comorbidities such as intellectual disability (ID), attention deficit hyperactivity disorder (ADHD), and physical conditions [4]. ID is common and important among children with ASD. In the US, 35.2% of the 8-year-old children with ASD had intelligence quotient (IQ) scores of 70 or lower, and 23.1% had borderline scores [2]. The Diagnostic and Statistical Manuel of Mental Disorders, Fifth Edition (DSM-5) stated that the IQ subitems of individuals with ASD and ID are unbalanced. Unlike their nonverbal skills, their social communication and interaction skills (e.g., nonverbal problem solving, fine motor skills) are significantly impaired [5].

ID may adversely affect the social interaction and behavior of individuals with autism. The greatest difficulty of children with ASD and ID is in developing social competence [6]. Lower intelligence is also related to lower adaptive function [7]. Furthermore, differences in IQ might stand behind the variations in ADHD in children with ASD. The severity of attention deficit and impulsivity might be related to the IQ level more than to the autism severity [8]. Moreover, low intelligence levels of children with ASD could negatively affect their long-term prognosis. Only about a quarter of the individuals with autism and an average intelligence level live independently in the US, while the rest remain with their families to late adulthood or beyond [9]. Premature mortality in individuals with ASD and ID is higher than those without ID [10, 11]. Therefore, low cognition function affects ASD symptoms and reduces the quality of life of these individuals. These associations highlight the severity of the cooccurrence of ID and ASD.

Early childhood is characterized by brain plasticity and neurodevelopmental instability. When an individual under the age of 5 years fails to meet the expected intellectual function developmental milestones, a global developmental delay (DD) will be diagnosed rather than ID [5]. Neurodevelopment or intelligence level evaluation is important for early ASD detection, accurate diagnosis, and precise intervention. Autistic symptoms usually appear around the age of 12–24 months but might be detected earlier in severe DD cases or later in mild ones [5]. It was reported that children with ASD and IQ ≤ 70 were more likely to be diagnosed by the age of three than those with IQ > 70 [2]. Hence, evaluation of the neurodevelopmental level of children at an early age could help identify and accurately diagnose ASD. Additionally, cognitive delays in children with ASD could lead them to develop greater deficits in adaptive functioning, social skills, and disruptive behavior and show limited neurodevelopmental progress, even following rigorous intervention [12, 13]. These findings suggested that such children require more intensive or adaptive interventions as they might respond poorly to the standard ASD-specific ones. Therefore, earlier developmental assessment in children with ASD would help design appropriate interventions earlier. Pierce Karen et al. reported that 84% of toddlers diagnosed with ASD at their first visit retained this diagnosis at the age of 3–4 years [14]. Besides, most children diagnosed with poor-functioning ASD (96%) had ASD at follow-up [15]. This suggests that pediatricians specializing in neurodevelopmental behavior can diagnose ASD at a young age.

To date, no multi-center large-scale study analyzed the characteristics of preschool children with ASD and ID or the association between their neurodevelopmental levels and core symptoms in China. Hence, we conducted this large-scale, multi-center cross-sectional study. We aimed to investigate the distribution of neurodevelopmental characteristics in preschool children with ASD in China and explore the relationship between their neurodevelopmental levels and core symptoms. Our study could improve our understanding of these features in this young population and provide a useful reference for early identification, diagnosis, and accurate intervention in ASD.

## Methods

### Study population

**Fig. 1:**
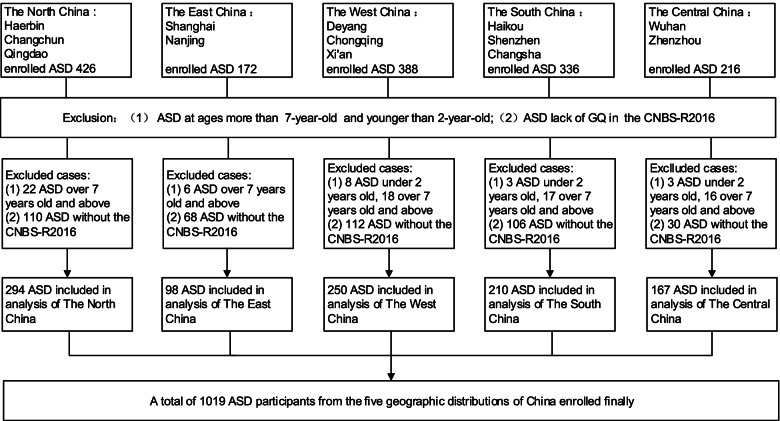
Flowchart of the participant recruitment process in this multi-center study in China. ASD, autism spectrum disorder; CNBS-R2016, Children Neuropsychological and Behavior Scale-Revision 2016

This study is part of the China Multi-Center Preschool Autism Project (CMPAP) [16]. Participants were recruited between May 2018 and December 2019 from 13 cities around China. Details about the participating centers and the subject enrollment process are presented in Fig. 1. The study enrolled 1538 children with ASD from outpatient clinics and special education institutions. After excluding 93 children outside the inclusion age range (2–7 years) and 426 due to missing data or assessment by the Gesell Developmental Schedules (GDS) instead of the Children Neuropsychological and Behavior Scale-Revision 2016 (CNBS-R2016), the study included 1019 children with ASD. A psychologist and a pediatrician with expertise in neurodevelopmental behavior diagnosed ASD in the enrolled children at each of the participating centers following the criteria of Diagnostic and Statistical Manual of Mental Disorders, 5th Edition (DSM-5) [5].

Participation was voluntary, and the primary caregiver of all participants signed informed consent forms. The Ethics Committee of the Children’s Hospital of Chongqing Medical University approved the study [Number: (2018) IRB (STUDY) NO. 121], and the study was registered in the Chinese Clinical Trial Registry (ChiCTR; Registration number: ChiCTR2000031194).

### Neurodevelopment and core symptom assessment in preschool children with ASD

The caregivers of the participants completed a questionnaire that included the name, age, sex, and ethnicity of the patient, and parental education status. The ASD symptoms were evaluated using the Autism Behavior Checklist (ABC), Social Responsiveness Scale (SRS), and Childhood Autism Rating Scale (CARS). The ABC assesses autism-related behavioral problems using five subscales: sensory, relating, stereotypic behavior, language, and social independence, with the score for normal children < 53 points [17]. The SRS uses five subscales to assess autism-related social impairment: social awareness, communication, and motivation, and autistic mannerisms. Typically-developing children score < 65 points [18]. The CARS is a developmental pediatrician observation scale that assesses autism symptom severity. It has 15 items, each rated on a four-point scale. Children with mild-to-moderate autism score 30–36 points, and those with severe autism score over 36 points [17]. The CNBS-R2016 assessed the neurodevelopment of the children. It was developed by the Capital Institute of Pediatrics, China, as a diagnostic and evaluation tool to assess the neurodevelopmental level and autism symptoms. The six subscales of CNBS-R2016 are gross motor, fine motor, adaptive behavior, personal-social, language, and communication warning behavior (CWB), which indicates the risk of the impaired social communication often reported in autism. A score under seven points in the communication warning behavior subscale indicates normal development, a score of 7–12 points suggests that follow-up is called for, a score of 12–30 points suggest the possible presence of a communication and interaction disorder, and a score higher than 30 points suggests a possible diagnosis of ASD. DD was determined when the general developmental quotient (GQ) and subscale developmental quotients (DQs) of Gross Motor,Fine Motor,Language and Personal-Social domains were under 70 points [under two standard deviations (SDs)]. The DD level can be mild (GQ, 55–69) or moderate and below (GQ ≤ 54) [19]. Assessment of the development of children with ASD by the CNBS-R2016 and Griffiths Mental Development Scales-Chinese (GDS-C) was shown to be consistent [19]. The number of valid assessments by the ABC, SRS, CARS, and CNBS-R2016 were 977, 883, 891, and 1019, respectively.

### Statistical analysis

The Shapiro–Wilk test was used to assess data normality. Continuous variables are presented as means ± standard deviations (M ± SD). Categorical variables are presented as counts (%) and were compared using the chi-squared test, followed by the Bonferroni correction for multiple comparisons. Missing values in the ABC, SRS, and CARS scores were substituted by multiple imputation. The DQs in the CNBS-R2016, ABC, SRS and CARS had skewed distribution, so the Kruskal-Wallis test compared the data, and the results are presented as medians and interquartile ranges (IQRs). Spearman’s rank correlation coefficient explored the association between the neurodevelopmental levels and core symptoms in children with ASD. Statistical analysis was performed using the IBM SPSS Statistics for Windows, Version 19.0 (IBM Corp., Armonk, NY, USA). Figures were generated using the GraphPad Prism software, Version 8.0.2.263 (GraphPad Software, San Diego, CA, USA). A *p*-value < 0.05 was considered statistically significant.

## Results

### Participant characteristics

The participant characteristics are presented in Table 1. Of the enrolled 1019 preschoolers with ASD, 835 were boys and 184 girls (male-to-female ratio, 4.54:1) at a mean age of 4.12 (2.00–6.98) years. Of these children, 93.2% were of the Han ethnicity and the remaining 6.8% of other ethnicities. Parental education levels included high school or below (48.0% of mothers and 42.3% of fathers) and college or above (52.0% of mothers and 57.7% of fathers) (Table 1).

**Table 1:** Participant characteristics (*n* = 1019)

Characteristic	ASD
Age (years), mean ± SD (range)	4.12 ± 1.17 (2 ~ 6.98)
Sex, n (%)	
Male	835(81.9)
Female	184(18.1)
Ethnicity, n (%)	
Han	950(93.2)
Others	69(6.8)
Maternal education level, n (%)	
High school or below	489(48.0)
College or above	530(52.0)
Paternal education level, n (%)	
High school or less	431(42.3)
College or above	588(57.7)

*ASD* autism spectrum disorder; *SD* standard deviation

### Prevalence of DD among children with ASD

A GQ under 70 indicates DD. Among the participating children, 322 (31.6%) were at a normal neurodevelopmental level (GQ ≥ 70), and 697 (68.4%) showed DD (GQ < 70). Of these, the DD of 333 (32.7%) was mild (GQ, 55–69), and it was moderate (GQ, 40–54) in 269 (26.4%), and severe and extremely severe (GQ ≤ 39) in 95 (9.3%). Overall, the prevalence of DD in our cohort was 68.4%, of which most (32.7%) showed mild DD (Table 2).

**Table 2:** Prevalence of intellectual developmental delay among children with ASD

Level of neurodevelopment	*n*	Rate(%)
Normal (GQ ≥ 70)	322	31.6%
Developmental delay (GQ < 70)	697	68.4%
Mild delay (GQ, 55–69)	333	32.7%
Moderate delay (GQ, 40–54)	269	26.4%
Severe delay (GQ ≤ 39)	95	9.3%

*ASD* autism spectrum disorder; *GQ* general quotient

### Uneven or unbalanced neurodevelopmental domains in children with ASD

The DD in gross motor, fine motor, language, and personal-social skills in children with ASD are shown in Table 3. The highest DD rates were in language (73.7%) and personal-social (77.0%) skills, followed by fine motor (68.9%) and gross motor (34.0%) skills (respectively, *p* < 0.001). The DQ of the gross motor skill (median, 79) was the highest, followed by fine motor (median, 60), personal-social (median, 54), and language (median, 49) skills (respectively, *p* < 0.001). The DQ of the fine motor skills was significantly higher than of the language and personal-social skills (respectively, *p* < 0.001).

**Table 3:** DQ characteristics in the four developmental subscales

Skill item	DQ [Median (IQR)]	DQ ≥ 70 [n(%)]	DQ < 70 [n(%)]
Gross motor	79 (64,92)	673(66.0%)	346(34.0%)
Fine motor	60 (47,74)^a^	317(31.1%)*	702(68.9%)*
Language	49 (33,71)^ab^	268(26.3%)*	751(73.7%)*
Personal-social	54 (42,68)^abc^	234(23.0%)*^#^	785(77.0%)*^#^
H/χ2	695.723	523.089	
*p*-value	< 0.0001	< 0.0001	

^a^ = statistical difference when compared with gross motor DQ(*p < 0.001*); ^b^ = statistical difference when compared with fine motor DQ(*p < 0.001*); ^c^ = statistical difference when compared with language DQ(*p < 0.001*); * = statistical difference when compared with gross motor DQ(*p < 0.001*); ^#^ = statistical difference when compared with fine motor DQ(*p < 0.001*)

Categorical variables were compared using the chi-squared test followed by Bonferroni correction for multiple comparisons. Due to their skewed distribution, the four developmental subscales were compared by the Kruskal-Wallis test

*DQ* developmental quotient; *IQR* interquartile range

We divided the participants into three subgroups based on their GQ: no DD, mild DD, and moderate and below DD. As can be noted from Table 4, the groups showed a similar trend, with the highest DQ in gross motor skills, followed by fine motor, language, and personal-social skills. The DQ of the fine motor skill was statistically similar to the language and personal-social skills in the no DD group (*p = 0.14*, 1.00, respectively) but significantly higher in the other two groups (*p* < 0.001). These findings indicate that the neurodevelopmental level structure in preschool children with ASD is uneven regardless of whether they had DD or not. Besides, language and personal-social skill development was most affected, especially in those with DD, followed by fine motor skills. Gross motor development was the least affected.

**Table 4:** DQ characteristics in children with ASD with and without DD

Item	No DD [Median (IQR)] (*n* = 322)	Mild DD [Median (IQR)] (*n* = 333)	Moderate and below DD [Median (IQR)] (*n* = 364)
Gross motor	96 (87,106)	79 (69,88)	61 (52,72)
Fine motor	79.5 (70,91)^a^	61 (54,68)^a^	44 (35.25,51)^a^
Language	83 (68,96)^a^	51 (40,60)^ab^	30 (23,38.75)^ab^
Personal-social	76 (65.75,87.25)^ac^	54 (49,61)^abc^	38 (32,45)^abc^
H	211.983	565.304	630.603
*p*-value	< 0.001	< 0.001	< 0.001

^a^ = statistical difference when compared with gross motor DQ (*p < 0.001*)*;* ^b^ = statistical difference when compared with fine motor DQ(*p < 0.001*); ^c^ = statistical difference when compared with language DQ(*p < 0.001*)

Due to their skewed distribution, the four developmental subscales were compared by the Kruskal-Wallis test

*DQ* developmental quotient; *ASD* autism spectrum disorder; *DD* developmental delay; *IQR* interquartile range

### Associations between neurodevelopment quotients and core symptoms in preschool children with ASD

To explore the associations between the neurodevelopment levels and core symptoms in children with ASD, we compared the ABC, SRS, CARS, and CWB scores in the CNBS-R2016 of the no DD, mild DD, and moderate and below DD groups. We found that the moderate and below DD group had the highest ABC, SRS, CARS, and CWB scores, followed by the mild DD and no DD groups (Table 5).

**Table 5:** Comparison of the ABC, SRS, CARS, CWB scores in ASD children with various neurodevelopmental levels

	No DD (*n* = 322)	Mild DD (*n* = 333)	Moderate and below DD (*n* = 364)	H/F	*p*-value
ABC	39 (25,55)	50 (37,66.5)*	59 (40,71)*#	378.839	< 0.001
SRS	81 (65,97)	93 (78,109.2) *	96 (82,112)*#	360.469	< 0.001
CARS	28 (25,32)	33 (29,37)*	35 (31,41)*#	972.986	< 0.001
CWB	33 (17,49)	42 (28,58)*	52 (34,66)*#	401.315	< 0.001

* = statistical difference when compared vs. children with ASD of normal neurodevelopmental level (*p < 0.001*); # = statistical difference when compared vs. children with ASD and a mild developmental delay (*p < 0.001*)

The distributions of the ABC, CARS, SRS, and CWB scores in the three groups were skewed, so the Kruskal-Wallis test was used

*ABC* Autism Behavior Checklist; *SRS* Social Responsiveness Scale; *CARS* Childhood Autism Rating Scale; *CWB* Communication warning behavior; *ASD* autism spectrum disorder; *DD* developmental delay

**Fig. 2:**
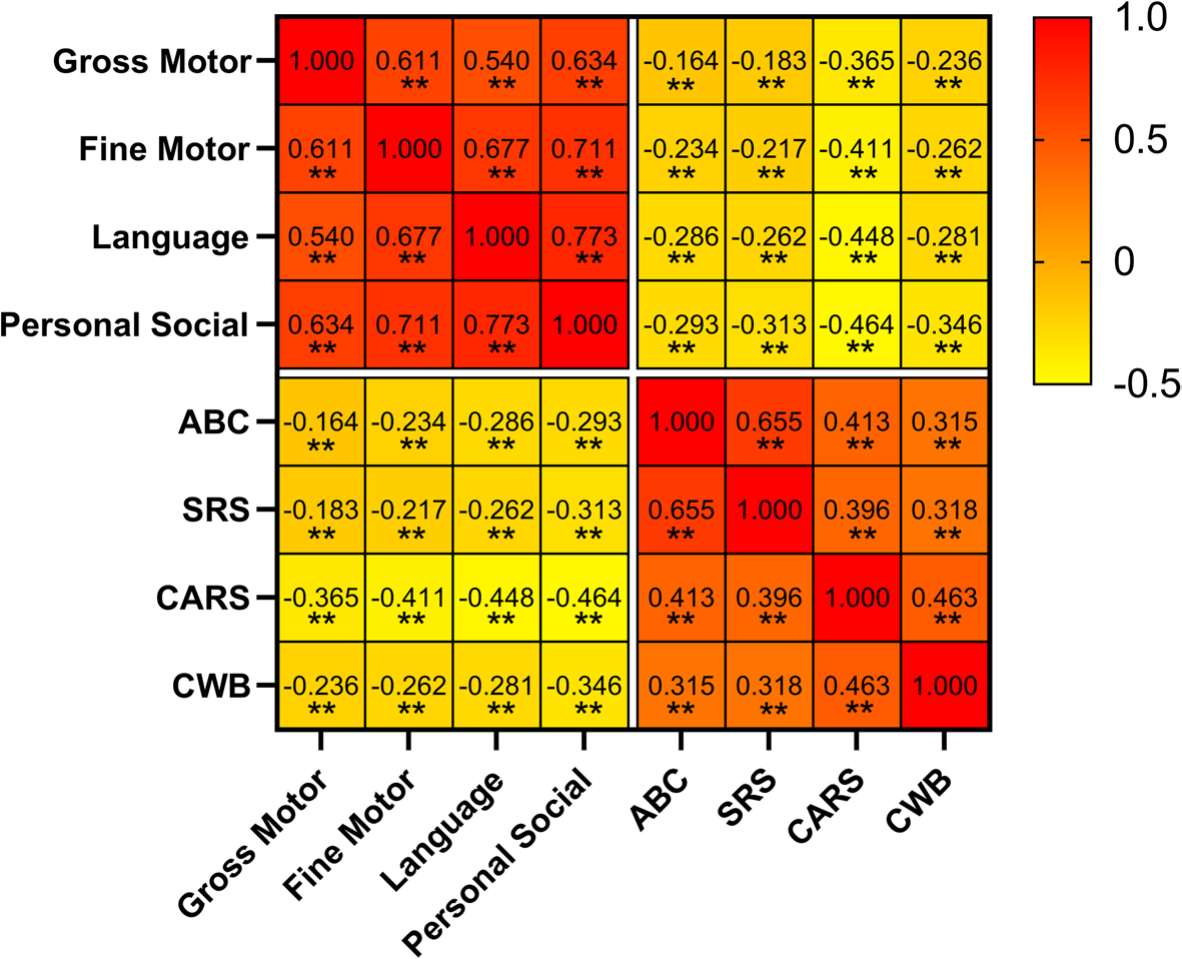
Heat map showing the correlation between four developmental subscales and core symptoms in ASD children. **p < 0.01

Spearman’s rank correlation coefficient (rs) analyzed the associations between the neurodevelopment quotients of the four subscales (gross motor, fine motor, language, and personal-social) and the ABC, SRS, CARS, and CWB scores, shown as a correlation heat map in Fig. 2. Orange in the heat map indicates positive correlation, and yellow indicates negative correlation. It can be seen from the heat map that all development levels were negatively correlated with the scores in all scales but were mutually positively correlated among them. The correlations between the DQ of the four neurodevelopmental levels and the scores in the four scales (all *p* < 0.01) included language (rs_ABC_ = − 0.286, rs_SRS_ = − 0.262, rs_CARS_ = − 0.448, and rs_CWB_ = − 0.281) and personal-social (rs_ABC_ = − 0.293, rs_SRS_ = − 0.313, rs_CARS_ = − 0.464, and rs_CWB_ = − 0.346) as the strongest, followed by fine motor (rs_ABC_ = − 0.234, rs_SRS_ = − 0.217, rs_CARS_ = − 0.411, and rs_CWB_ = − 0.262) and gross motor (rs_ABC_ = − 0.164, rs_SRS_ = − 0.183, rs_CARS_ = − 0.365, and rs_CWB_ = − 0.236). These results suggest that the lower the neurodevelopmental level the children with ASD had, the more severe the autism core symptoms were. The neurodevelopmental levels appear to be most strongly associated with the CARS scores.

## Discussion

ASD clinical heterogeneity is considerable, and it often co-exists with other diseases such as ID, which can adversely affect the symptoms, quality of life, and prognosis. However, few large-scale studies explored the neurodevelopmental characteristics of preschool children with ASD. This study investigated the characteristics of the neurodevelopmental domains and the association between them and autistic symptoms in preschool children with ASD.

Intellectual status assessment in children with ASD is critical. For example, the DSM-5 requires clarifying if ASD is associated with ID [5]. The prevalence of ID in individuals with ASD varied greatly among studies. A large-sample study from 2003 showed that 68% of the 3–10-year-old children with ASD had ID [20]. Baird et al. reported that 55% of the 9–10-year-old children with ASD had IQ < 70 and that the highest prevalence of ID (73%) was associated with narrow autism [21]. Watfa et al. showed that the IQ of individuals with ASD had a U-shaped association with age, with prevalence of ASD in combination with ID at 56, 76, and 56% in children aged 3–5, 6–8, and 9–13 years, respectively [22]. In the US, among 8-year-old children with ASD, 35.2% were classified as having ID, and 23.1% as being in the borderline range [2]. Among the 2–7-year-old children with ASD in our study, more than half (68.1%) had DD (GQ < 70), mild in 32.6%, moderate in 26.2%, and severe in 9.2%. The rate of ASD with DD in our study was relatively high. This could be attributed to the special education institutions and outpatient clinics acting as the sources of our study participants. Some mild cases tend to go to regular schools and, therefore, were not investigated. Therefore, our sampling framework tended to be dominated by individuals with low IQ. Future research should cover children with ASD from all possible sources. Although some preschool children with ASD and mild to moderate ID can gradually improve in their neurodevelopmental level with age [23], they could be classified as having DD when young. The rate of DD in our study was relatively high because we focused on preschool children.

Many studies have consistently shown that ASD was associated with an irregular intellectual profile. It scored high on the Perceptual Reasoning Index and low on the Verbal Comprehension Index, Working Memory Index, and Processing Speed Index [24, 25]. In general, the non-verbal IQ of children with ASD was higher than their verbal IQ [26, 27]. We also found uneven neurodevelopment domains in the preschool children with ASD. Language and personal-social skills showed high rates of DD, followed by fine motor and gross motor skills. We subdivided the children based on their GQ into no DD, mild DD, and moderate and below DD groups and found that the fine motor, language, and personal-social developmental skills were significantly lower than gross motor in all three groups. Furthermore, the fine motor skill was statistically similar to the language and personal-social skills in the no DD group but significantly higher in the other two groups. These findings suggest that language and personal-social development impairment were most prominent among children with low-functioning ASD. Our results are consistent with the differential diagnosis of ASD in individuals with ID stated in the DSM-5: “A diagnosis of ASD in an individual with ID is appropriate when social communication and interaction are significantly impaired relative to the developmental level of the individual’s nonverbal skills (e.g., fine motor skills, nonverbal problem solving)” [5].

The ABC, SRS, CARS, and CWB scores in the no DD group in our study were the lowest, followed by mild DD and moderate and below DD. This was consistent with reports showing that individuals with ASD and low mental age exhibit more severe symptoms than the children with ASD and high mental age [15, 28]. Hinnebusch et al. reported that children with low-functioning ASD maintained their diagnosis and showed more severe symptoms and lower neurodevelopmental progress than the children with ASD [15]. Therefore, clinicians should strive to make an early and accurate diagnosis of these children to initiate early intervention. Correlation analysis showed that the DQ values of the four neurodevelopmental domains were negatively correlated with the ABC, SRS, CARS, and CWB scores, of which language and personal-social skills showed the strongest correlations. Similarly, a study of 222 5-year-old children with pervasive developmental disorders showed that the language skill level was positively correlated with psychological development and symptom intensity. The more severe the symptoms in children with ASD, the more apparent was their neurodevelopmental delay, and the more serious their language problems were [29]. Bennett et al. have demonstrated that children with ASD and language impairment were significantly more socially impaired than those with ASD alone [6]. Some studies have indicated that the motor skills of children with ASD were related to their social skills and that children with ASD and lower social and communication functions showed more significant impairments in their dexterity and fine motor skills. Similar to previous studies, our study found that the four neurodevelopmental domains were mutually positively correlated. LeBarton and Landa reported that early delay in motor skill development might be related to a delay in language skill development [30, 31]. Besides, it was shown that the fine motor skills in six-month-old infants could predict the results of their expressive language when they reach the age of 3 years [32]. As a whole, our study showed a negative association between the neurodevelopmental level of children with ASD and their autism symptoms, with a mutual association among the neurodevelopmental domains. Therefore, an early and comprehensive developmental evaluation in children with ASD is of great importance, as it could help develop personalized intervention plans for these children.

Our study had several limitations. First, our participants came from special education institutions and outpatient clinics, restricting our ability to enroll children with ASD in the entire DQ spectrum. Therefore, the rate of ASD with DD in this study was relatively high. Second, this was a cross-sectional study; therefore, we cannot indicate how the DQ and core symptom profiles changed through childhood.

## Conclusion

DD was detected in 68.4% of the participating preschool children with ASD, showing an uneven pattern in the neurodevelopment domains. The DD in the language and personal-social skills were the worst, followed by fine motor, with gross motor being the least affected skill. The neurodevelopmental levels were negatively associated with core autism symptoms, of which language and personal-social skills had the strongest correlations. These results suggest that a comprehensive neurodevelopmental level assessment should be part of the regular evaluation process of patients with ASD visiting the clinic, and long-term follow-up should be enacted. These could promote early diagnosis and intervention and help develop personalized intervention plans for children with ASD.

## Data Availability

Datasets generated during the current study are not publically available due to ethical restrictions but are available from the corresponding author on reasonable request.

## Abbreviations

ASD: Autism spectrum disorder
CNBS-R2016: the revised Children Neuropsychological and Behavior Scale
SRS: Social Responsiveness Scale
ABC: Autism Behavior Checklist
CARS: Child Autism Rating Scale
CWB: Communication warning behavior
GQ: General quotient
DQ: Developmental quotient
DD: Developmental delay
ID: Intellectual disability
ADHD: Attention deficit hyperactivity disorder
IQ: Intelligence quotient
DSM-5: The Diagnostic and Statistical Manuel of Mental Disorders, Fifth Edition
CMPAP: The China Multi-Center Preschool Autism Project
GDS: The Gesell Developmental Schedules
IQRs: Interquartile ranges;rs:rank correlation coefficient
